# Deep Learning-Based Method for Compound Identification in NMR Spectra of Mixtures

**DOI:** 10.3390/molecules27123653

**Published:** 2022-06-07

**Authors:** Weiwei Wei, Yuxuan Liao, Yufei Wang, Shaoqi Wang, Wen Du, Hongmei Lu, Bo Kong, Huawu Yang, Zhimin Zhang

**Affiliations:** 1 Technology Center, China Tobacco Hunan Industrial Co., Ltd., Changsha 410014, China; weiww0216@hngytobacco.com (W.W.); duwen0621@hngytobacco.com (W.D.); 2 College of Chemistry and Chemical Engineering, Central South University, Changsha 410083, China; 212311021@csu.edu.cn (Y.L.); wyf991314@163.com (Y.W.); 8202180822@csu.edu.cn (S.W.); hongmeilu@csu.edu.cn (H.L.); 3 Flavors and Fragrances Research Institute, Technology Center, China Tobacco Hunan Industrial Co., Ltd., Changsha 410014, China

**Keywords:** deep learning, identification, NMR, mixture analysis

## Abstract

Nuclear magnetic resonance (NMR) spectroscopy is highly unbiased and reproducible, which provides us a powerful tool to analyze mixtures consisting of small molecules. However, the compound identification in NMR spectra of mixtures is highly challenging because of chemical shift variations of the same compound in different mixtures and peak overlapping among molecules. Here, we present a pseudo-Siamese convolutional neural network method (pSCNN) to identify compounds in mixtures for NMR spectroscopy. A data augmentation method was implemented for the superposition of several NMR spectra sampled from a spectral database with random noises. The augmented dataset was split and used to train, validate and test the pSCNN model. Two experimental NMR datasets (flavor mixtures and additional flavor mixture) were acquired to benchmark its performance in real applications. The results show that the proposed method can achieve good performances in the augmented test set (ACC = 99.80%, TPR = 99.70% and FPR = 0.10%), the flavor mixtures dataset (ACC = 97.62%, TPR = 96.44% and FPR = 2.29%) and the additional flavor mixture dataset (ACC = 91.67%, TPR = 100.00% and FPR = 10.53%). We have demonstrated that the translational invariance of convolutional neural networks can solve the chemical shift variation problem in NMR spectra. In summary, pSCNN is an off-the-shelf method to identify compounds in mixtures for NMR spectroscopy because of its accuracy in compound identification and robustness to chemical shift variation.

## 1. Introduction

The main technologies for analyzing mixtures consisting of small molecules include nuclear magnetic resonance (NMR) and mass spectrometry (MS). Each has its advantages and disadvantages [1] concerning sensitivity and reproducibility. NMR is reproducible and nondestructive, but its sensitivity is relatively poor, whereas MS is highly sensitive, it shows low reproducibility [2]. Recently, technologies such as low temperature probes and high-field NMR spectrometers have achieved large improvements in the sensitivity of NMR [3]. By probing local magnetic fields surrounding specific atomic nuclei, NMR can measure signals with the electronic structures and functional groups information from molecules. Therefore, NMR is particularly useful for identifying the structures of small molecules [4,5]. Furthermore, the advantages of nondestructive, unbiased and easy sample preparation make NMR spectroscopy widely used in many fields, including chemistry [6], metabolomics [7,8,9], drug discovery [10,11,12], food [13,14], natural products [15,16], flavors [17], environments [18], forensic [19], cultural heritage [20], etc. It is mainly used for three tasks: identification, verification and quantification [21]. For the identification and verification tasks, it is necessary to evaluate the similarities between NMR spectra. Similarity methods for NMR spectra can be divided into two categories (chemical shift similarity and spectral similarity), according to their inputs [22]. The inputs of chemical shift similarity-based methods are peak tables, which are commonly used for searching NMR spectral databases [23,24,25]. The inputs of spectral similarity-based methods are full NMR spectra, which are used to calculate the vector-based similarity or distance [26]. The traditional similarity methods may fail in real applications because of chemical shift variations [27]. To avoid the chemical shift variation problem in NMR spectroscopy, the commonly used strategies are binning [28,29,30], shift alignment [31,32,33] and shift-insensitive similarities [34,35]. They are successfully used for the identification of pure substances and the verification of complex samples. In many cases, mixtures of small molecules are common in chemistry, and they can be analyzed by NMR spectroscopy directly without further separation and purification [36,37]. Due to the signal overlap and interferences in NMR spectra, the previously mentioned similarity methods may fail in analyzing the mixtures. Therefore, the identification of components in mixtures is highly challenging. One category of methods acquires 2D NMR spectra (e.g., TOCSY) and decomposes them using deconvolution methods to obtain the 1D NMR spectra of components for identification [38,39,40,41,42]. The other category of methods uses statistics, chemometrics and pattern recognition algorithms to identify components directly from NMR signals [43,44,45,46,47]. Furthermore, the combining MS, NMR and algorithm delivers good results for the reliable identification of the constituents in complex mixtures [48].

Deep learning is a category of flexible machine learning methods based on neural networks with multiple hidden layers to learn multilevel representation automatically for specific tasks [49]. It has three distinct advantages over the traditional learning methods. First, their network architectures are flexible enough to handle various kinds of raw inputs directly. For instance, there are convolutional neural networks (CNN) for computer vision [50], recurrent neural networks (RNN) [51], attention networks [52] for natural language processing and graph neural networks (GNN) for graph data structures [53]. Second, the multiple hidden layers can automatically transform the raw inputs into multilevel representations using a general purpose learning procedure [54]. Third, the deep learning methods have high expressive power and model capacity because of the depth efficiency, which can take full advantage of big data [55]. Due to these advantages, deep learning-based methods have achieved a state-of-the-art performance in numerous related fields of NMR spectroscopy [56,57], ranging from spectral reconstruction [58,59,60], denoising [61], peak picking [62,63], chemical shift prediction [64,65,66,67,68] and molecular recognition (the SMART method proposed by Zhang et al. in 2017) [69] to molecule identification [70,71,72]. It has shown unprecedented capabilities in solving difficult problems in NMR spectroscopy.

In this study, a pseudo-Siamese convolutional neural network (pSCNN) for NMR spectroscopy was developed to solve the chemical shift variation and the signal overlap problems in the component identification of mixtures inspired by Siamese neural networks [73,74] and the DeepCID method [75]. Each input of pSCNN is a spectral pair consisting of two full ^1^H NMR spectra: one is the spectrum of a pure compound from a spectral database, and the other is the spectrum of a mixture. The spectrum of a pure compound and the spectrum of a mixture are fed into two independent subnetworks consisting of convolutional layers, respectively. The translation-invariant representations can be learned for subsequent comparisons. The learned representations of the pure compound and the mixture are concatenated and fed into dense layers to predict their inclusion relationship (whether the mixture includes the pure compound or not). A data augmentation procedure was implemented to generate both positive and negative inputs for training, validating and testing the pSCNN model. The hyperparameters were optimized to obtain reasonable architecture and better performances. The compound identification procedure was developed by predicting the inputs of each pure compound in the spectral database and the mixture with the model. The NMR spectra of known flavor mixtures and additional flavor mixture were acquired by an NMR spectrometer to benchmark the performance of the proposed method. Finally, the translational invariance of CNN was demonstrated to be suitable for solving the chemical shift variation problem when identifying compounds in a mixture with NMR spectroscopy. The main novelties of pSCNN compared to the SMART method are: (1) the two subnetworks of pSCNN have the same architecture, but their weights are trained separately, whereas the subnetworks of SMART have the same weights, and (2) the pSCNN method acquires 1D NMR spectra, while SMART needs 2D NMR spectra. The acquisition of 2D NMR spectra is time-consuming and highly expensive. To the best of our knowledge, this is the first work on the comparison of 1D NMR by the pseudo-Siamese convolutional neural network.

## 2. Method

**Figure 1:**
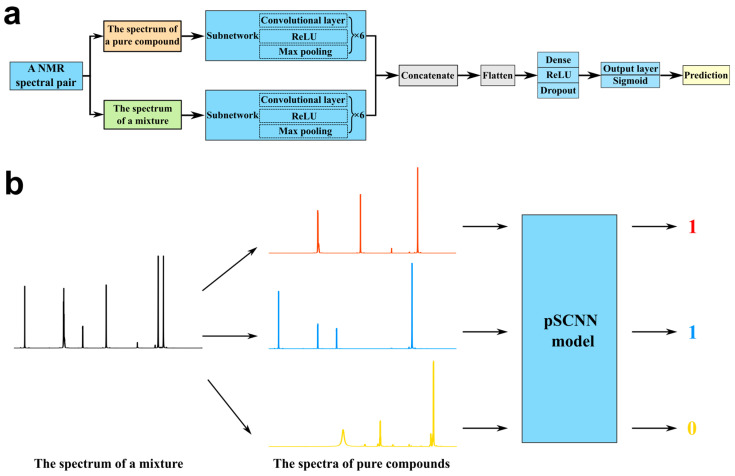
Schematic diagram of the proposed pSCNN method. (**a**) The network architecture of the pSCNN model. pSCNN consists of two subnetworks. Each subnetwork consists of six convolutional layers. The extracted features are concatenated and fed into two dense layers for prediction. (**b**) pSCNN model-based component identification. The inclusion relationship between each compound in the database and a mixture is predicted by the pSCNN model.

The schematic diagram of the proposed pSCNN method is shown in Figure 1. Its source code is available at https://www.github.com/yuxuanliao/pSCNN (accessed on 4 May 2022). It mainly consists of two parts: a pseudo-Siamese convolutional neural network and model-based component identification. The data augmentation procedure is essentially the superposition several NMR spectra sampled from a spectral database at random ratios with random noises. Both positive and negative spectral pairs are generated and partitioned into training, validation and test sets. The pSCNN takes an NMR spectral pair as its input, extracts high-level representations using CNN layers and predicts the probability of a pure compound in a mixture using dense layers. For each compound in the database, the model-based component identification predicts the probability of it in a mixture. The possible components of the mixture can be obtained by filtering the predicted probabilities using a threshold value. In the subsequent subsections, we will elucidate the principle of each part of this method as clearly as possible.

### 2.1. Data Augmentation

The data augmentation and samples split are shown in Figure S1. Due to the good additivity in the NMR spectra between the mixture and its components, a data augmentation method has been developed, which can be used to generate enough spectra for training, validating and testing the pSCNN model. Essentially, data augmentation is the superposition of several flavor standards randomly sampled from the database at random ratios. Since spectra acquired by an NMR spectrometer contain noises, some random noises should be generated and added into the augmented spectra. Here, several NMR spectra were randomly selected from the spectral database, and the spectrum of a specific component was chosen as the pure spectrum in the spectral pair. The spectrum of this specific component was superposed with the other sampled ones at random ratios in a given range (e.g., 0.2–1.0) to generate positive ones. Negative ones were generated in the same way without the spectrum of this specific compound. Noises were added into the spectra of both positive and negative ones. The NMR spectral pair of each augmented sample was obtained by combining the augmented spectrum with the spectrum of the specific compound. A total of 22,000 augmented NMR spectral pairs were generated. The augmented dataset was split into the training set (18,000 spectral pairs), validation set (2000 spectral pairs) and test set (2000 spectral pairs) randomly. In summary, the augmented dataset was generated for training the pSCNN model, optimizing the hyperparameters of pSCNN and evaluating its accuracy, sensitivity and specificity.

### 2.2. Convolutional Neural Network

CNN is a popular category of deep learning methods in computer vision. Recently, it has also been widely used in chemistry [76], especially analytical chemistry [77]. It basically consists of an input layer, convolutional layers, pooling layers, dense layers and an output layer. Convolutional layers directly learn the multiple-level and translation-invariant representations from the raw inputs. Pooling layers reduce the dimensionalities of the data and improve the computational efficiency by combining several adjacent features. Dense layers perform nonlinear combinations of higher-level representations to achieve specific classification or regression tasks. The output layer is often a special dense layer, with its output size equaling the number of labels that each input has.

The core of the convolutional layer is convolution kernels, which are filters of a set of trainable parameters. Each convolution kernel scans the input with a given stride to extract features as its output. A feature map is generated by detecting a similar feature at different locations with the same convolution kernel. The most significant advantages of the convolutional layer are sparse connectivity and parameter sharing. Sparse connectivity is a learning process from local to global, which gradually enhances the understanding of global information with less parameters. Parameter sharing means that the same convolution kernel scans the feature map with a given stride, which can reduce the number of parameters significantly. The one-dimensional convolution layer is defined as follows:

(1) $$out_{N_{i},C_{out_{j}}}=f(bias_{C_{out_{j}}}+weight_{C_{out_{j}}}⊗input_{N_{i}})$$

where ⊗ is the cross-correlation operation, $C_{out_{j}}$ is the number of channels, $N_{i}$ is the batch size, f() is the activation function and the rectified linear unit (ReLU, f(x)=max(0,x)) is used as the activation function in this study.

Pooling layers are often used to reduce the dimensions of feature maps by computing the maximal value or mean value of a small cluster. As a result, the number of parameters to be learned and the amount of computations are reduced significantly. It can also improve the translation invariance. Here, the max pooling layer was used in the pooling layers of the pSCNN model.

Each dense layer is connected to all the outputs of its previous layer. It mainly performs a nonlinear combination of higher-level features from convolutional layers to achieve a specific prediction task. Meanwhile, there is a dropout layer following each dense layer to avoid overfitting and improve the generalization ability. The output layer is also a dense layer, with the output size equaling one. Since the output is the probability of a component in the mixture, Sigmoid is used as the activation function in the output layer.

### 2.3. Pseudo-Siamese Convolutional Neural Network

Compared with a traditional neural network with one input, a Siamese neural network uses two subnetworks with the same architecture and weights to extract two comparable feature vectors from two different inputs. For identifying compounds in mixture-based NMR spectroscopy, two feature vectors are extracted from the NMR spectrum of a mixture and the NMR spectrum of a given compound, respectively. Then, these feature vectors are compared by dense layers to determine their inclusion relationship. Since the NMR spectra are from the mixture and the pure compound, it is not appropriate to use subnetworks with the same weights for extracting their feature vectors. In this study, two subnetworks have the same architecture, but their weights are trained separately. This type of neural networks is often called pseudo-Siamese neural networks, because the subnetworks do not share weights. Furthermore, the feature extraction subnetworks employ convolutional layers, so this neural network is called a pseudo-Siamese convolutional neural network. The learned feature vectors are concatenated and fed into the dense layers to identify whether the two inputs have an inclusion relationship, thus achieving compound identification in the NMR spectra of mixtures.

### 2.4. Architecture of pSCNN for NMR

**Figure 2:**
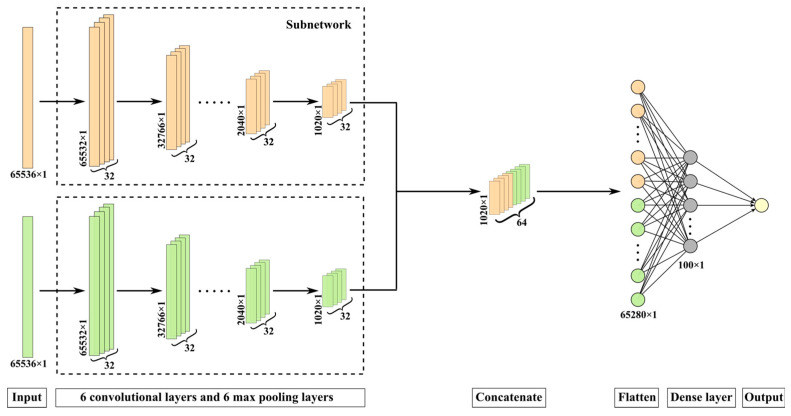
The detailed neural network architecture of pSCNN.

The detailed neural network architecture of pSCNN is shown in Figure 2. The input of pSCNN is an NMR spectral pair, which consists of one NMR spectrum of the mixture and one of the pure compound. The corresponding label of an NMR spectral pair is 0 or 1:1 if the mixture contains the pure compound and 0 otherwise. After feeding a spectral pair into pSCNN, their features are extracted by two subnetworks with the same architecture, respectively. Each subnetwork consists of 6 convolutional layers, each followed by a max pooling layer. The number of kernels for the convolutional layers is 32, and the kernel size is 5 × 1. The activation function of the convolutional layers is ReLU. They can learn the translation-invariant features from NMR spectra effectively, and the extracted features of two subnetworks are concatenated, flattened and fed into dense layers for comparison. The number of hidden units for the first dense layer is 100, and its activation function is also a ReLU. A dropout layer with the dropout rate equaling 0.2 is introduced to the dense layer for circumventing the overfitting problem. The output layer, the last dense layer, contains one hidden unit and uses the Sigmoid function as the activation function to form the final output. Binary cross entropy is chosen as the loss function, since it is suitable for binary classification problems. The Adam [78] optimizer is chosen as the optimizer because of its computational efficiency and little memory requirement.

### 2.5. Compound Identification with pSCNN

Given an NMR spectral database (**D**) and the NMR spectrum of a mixture (**x**), components in the mixture can be identified by the pSCNN model. The details of this identification method are described in the following procedure. Assuming that there are N NMR spectra of the standards in the NMR spectral database, the mixture spectrum is combined with these N spectra to form N spectral pairs $\left(D_{1},x),\cdots ,(D_{N},x\right)$. For each spectral pair, its probability is predicted by the pSCNN model. After predicting all N spectral pairs, the probabilities of all compounds in the database are obtained. The probabilities of these compounds are filtered by setting a threshold value (e.g., 0.5). The components with a predicted probability greater than this threshold are regarded as candidates in the mixture.

### 2.6. Evaluation Metrics

To evaluate the performance of pSCNN on the mixture analysis, the metrics used in this study are accuracy (*ACC*), true positive rate (*TPR*, sensitivity) and false positive rate (*FPR*). The mathematical formulas for *ACC*, *TPR* and *FPR* are as follows:

(2) $$ACC=\frac{TP+TN}{TP+TN+FP+FN}$$

(3) $$TPR=\frac{TP}{TP+FN}$$

(4) $$FPR=\frac{FP}{TN+FP}$$

where *TP*, *TN*, *FP* and *FN* are the number of true positives, true negatives, false positives and false negatives, respectively. Samples were labeled as positive or negative in binary classification. If both the prediction value and actual value are positive, the sample is *TP*. If both the prediction value and actual value are negative, the sample is *TN*. The sample is *FP* if the prediction value is positive and the actual value is negative or *FN* if the prediction value is negative and the actual value is positive.

## 3. Experiments

### 3.1. Flavor Standards

Deuterated dimethyl sulfoxide (DMSO-d_6_, >99.8 atom% D, contains 0.03% (*v*/*v*) TMS) was purchased from Ningbo Cuiying Chemical Technology Co., Ltd., Ningbo, China. A total of 24 flavor standards were purchased from Guangzhou Huafang tobacco flavor Co., Ltd., Guangzhou, China. The information of each flavor standard is listed in Table S1. The sample solution was produced by dissolving 250 μL of each flavor standard in 500 μL of DMSO-d_6_. Then, 600 μL of each sample solution were taken for NMR measurement. All ^1^H NMR spectra were acquired at 298 K on a Bruker AVANCE III 400 MHz NMR spectrometer (Bruker BioSpin, Rheinstetten, Germany). DMSO-d_6_ was used for the NMR field lock. TMS was used as the internal standard. The pulse program was chosen as zg30, and the number of scans was 16.

### 3.2. Known Flavor Mixtures

Two, three, four or five flavor standards were mixed randomly to form 15 mixtures with known components. The information of each flavor mixture is listed in Table S2. Each flavor mixture was prepared by taking 100 μL of each flavor standard and mixing them. The above prepared flavor mixture was added to 500 μL of DMSO-d_6_, then vortex-mixed for 1 min at room temperature and, finally, transferred 600 μL to an NMR tube for NMR measurements. The experimental conditions of the known flavor mixtures were set as those of the flavor standards.

### 3.3. Additional Flavor Mixture

The additional flavor mixture was provided by third-party personnel in the Technology Center of China Tobacco Hunan Industrial Co., Ltd., Changsha, China. The components of the additional flavor mixture were unknown when analyzing it with the pSCNN model. After submitting the predicted result to the Technology Center of China Tobacco Hunan Industrial Co., Ltd., we were informed of the corresponding components in this mixture. The information of the additional flavor mixture is listed in Table S3. The sample solution was prepared by dissolving 250 μL of the additional flavor mixture in 500 μL of DMSO-d_6_. Then, 600 μL of the sample solution was transferred into an NMR tube. As previously described, the same experimental conditions were used for NMR measurement.

## 4. Results and Discussion

### 4.1. Implementation and Computing Resources

In this study, the neural network and related modules were implemented in Python (version 3.8.13), Tensorflow GPU package (version 2.5.0) and scikit-learn (version 1.0.0). The NMR spectra were read into Python using the nmrglue package (version 0.8.dev0). The computing tasks were submitted to the Inspur TS10000 high-performance computing (HPC) cluster of Central South University using the Slurm workload manager (version 20.02.3). This HPC cluster has 1022 central processing unit (CPU) nodes, 10 fat nodes and 26 graphics processing unit (GPU) nodes. For the training of pSCNN models, it was a GPU node with 2 × Intel(R) Xeon(R) Gold 6248R processors, 2 × Nvidia Tesla V100s, 384G DDR4 memory and a CentOS 7.5 operating system.

### 4.2. Validation of Data Augmentation

**Figure 3:**
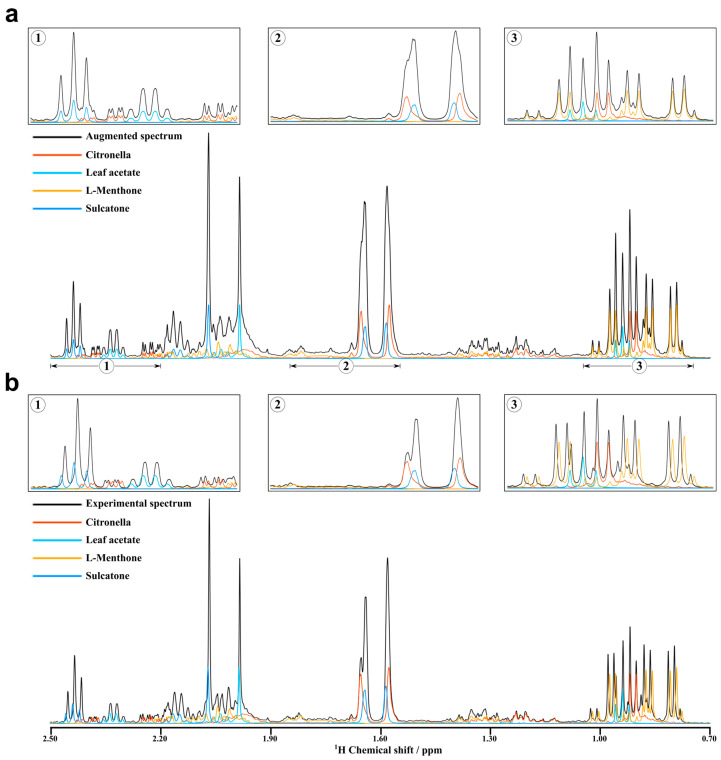
Augmented and experimental NMR spectra. (**a**) The spectrum of mixture obtained by data augmentation and its components spectra. (**b**) The experimental NMR spectra of the mixture and its components. (1–3) are the local zoomed-in views.

The augmented and experimental NMR spectra were compared to validate the rationality of the data augmentation. Here, the F10 mixture consisting of Sulcatone, L-Menthone, Citronellal and Leaf acetate was used as an example. An augmented spectrum was generated by the data augmentation method in Section 2.1 with the components of the F10 mixture and random ratios. Figure 3a,b show the augmented spectrum and the experimental spectrum of F10, respectively. The augmented spectrum is basically consistent with the experimental spectra from the local zoomed-in views, except for the chemical shift variations in the experimental spectra. Thus, it has shown that the data augmentation method can generate reasonable NMR spectra of mixtures from the NMR spectra of components.

### 4.3. Hyperparameters Optimization and Training

**Figure 4:**
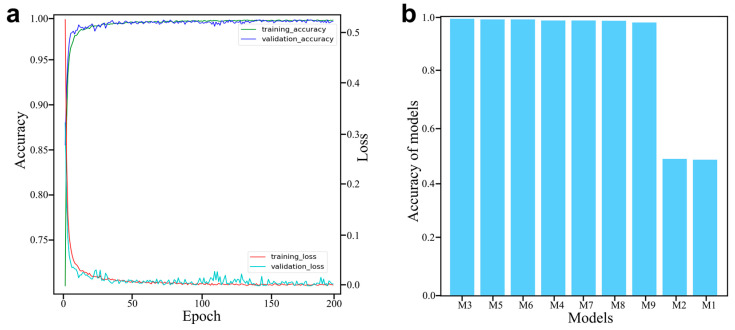
Optimization of the pSCNN model. (**a**) The accuracy curves and loss curves of training set and validation set. (**b**) The accuracy of different models on the validation set.

The optimization of the hyperparameters is crucial for establishing a high-performance model. For the pSCNN model used in this study, the key hyperparameters are the epoch, the learning rate and the number of convolutional layers, which should be optimized. Firstly, the epoch was set to 200, and the model was trained. The loss–epoch and accuracy–epoch curves are shown in Figure 4a. It can be seen that the model is basically stable after 100 epochs. Therefore, the epoch was set to 100. For the learning rate, the training is slow when too small, and the model does not converge when too large. Here, we investigated the learning rates in the range of 10^−2^ to 10^−5^. Combining the results in Table 1 and Figure 4b, it can be concluded that the model fails to converge with the learning rates in a range from 10^−2^ to 10^−3^. If the learning rate is set between 10^−4^ and 10^−5^, the model can be successfully trained. For the number of convolutional layers, we tested inside the range of 5–10. As can be seen in Table 1 and Figure 4b, the accuracy of the validation set increases and then decreases as the number of convolutional layers increases. The best performance was achieved when the number of layers was equal to 6. Therefore, the optimized epoch, learning rate and number of convolutional layers were 100, 10^−4^ and 6, respectively. The final model was chosen as M3, which could achieve an accuracy of 0.9990 on the validation set.

### 4.4. Performance Evaluation

**Figure 5:**
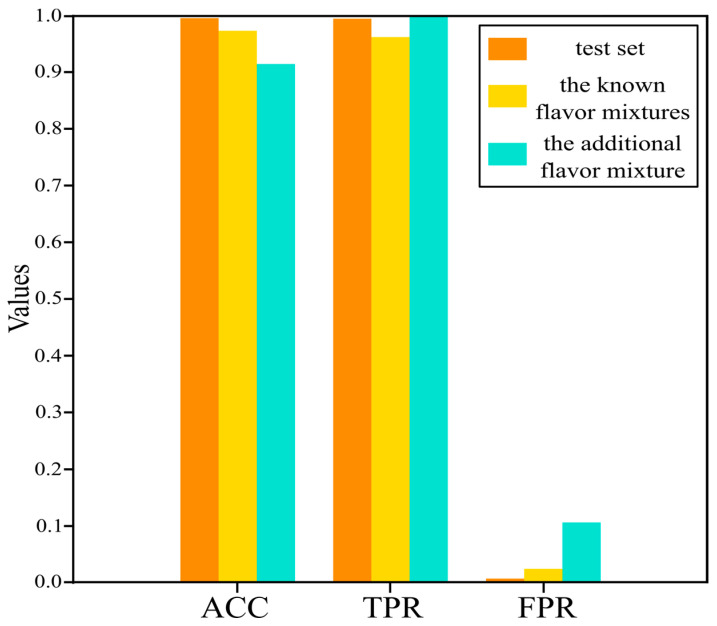
Performance evaluation on the test set and application of pSCNN on the known flavor mixtures and the additional flavor mixture.

The training set and the validation set have already been used to update the parameters and adjust the hyperparameters, respectively. The performance evaluation metrics obtained on them are often overoptimistic. To test the true performance of the model on unknown samples, an independent test set is usually used for a performance evaluation. Here, the test set in the augmented dataset was used to evaluate the performance of the pSCNN model on unseen samples. Each spectral pair in the test set was fed into pSCNN, and their features were extracted and transformed into the learned representations to predict the possibility of the pure compound in the mixture. There were 2000 spectral pairs in the test set. As shown in Figure 5, its ACC, TPR and FPR are 99.80%, 99.70% and 0.10% respectively, which guarantee the performance of the pSCNN model on unseen samples.

### 4.5. Results of Mixture Analysis

Due to its excellent performance on the test set, the pSCNN model was used to identify the flavor standards in the known flavor mixtures and the additional flavor mixture. For the known flavor mixtures, the components in each mixture are known. Therefore, they were used to verify the identification performance of pSCNN on real NMR spectra. The NMR spectrum of each mixture in the known flavor mixtures dataset was combined with the NMR spectra of flavor standards to form its spectral pairs. These spectral pairs were fed into the pSCNN model to predict the probabilities of the flavor standards in this flavor mixture. The results of the known flavor mixtures are shown in Figure 5 and Table 2. The detailed results of all mixtures in the flavor mixtures dataset are listed in Table S4. It can be seen that the ACC, TPR and FPR are 97.62%, 96.44% and 2.29%, respectively. Therefore, the performance of pSCNN for component identification in the mixtures was verified by analyzing the known flavor mixtures dataset.

It was further applied to analyze the unknown flavor mixtures in the additional flavor mixture dataset. Since the components of U1 were unknown when analyzing it with the pSCNN model, it can test the accuracy, sensitivity and specificity of pSCNN for real-world applications. It was predicted in the same way as for the mixtures in the known flavor mixtures. For the additional flavor mixture, the model-based prediction probabilities for the U1 mixture were ranked from high to low as β-Ionone, γ-Decalactone, γ-Nonanoic lactone, Citral, Leaf alcohol, Isovaleric acid and 2-Methylbutyric acid. After submitting the predicted candidates to the Technology Center of China Tobacco Hunan Industrial Co., Ltd., they sent us the real formulation of U1. The formulation of U1 is listed in Table S5. It can be seen that the predicted results of pSCNN match well with the formulation provided by the Technology Centre of China Tobacco Hunan Industrial Co., Ltd. The statistical results are also shown in Figure 5 and Table 2. The detailed results of the mixture in the additional flavor mixture dataset are listed in Table S5. The ACC, TPR and FPR on the additional flavor mixture dataset are 91.67%, 100.00% and 10.53%, respectively. The FPR in the additional flavor mixture (10.53%) is high, and the reasons are that Isovaleric acid and 2-Methylbutyric acid are structural isomers, and γ-Decalactone and γ-Nonanoic lactone are homologs, because their molecular structures differ by only one CH_2_ group. Actually, FPR is not an issue; this is because the false positives in the candidate components can be filtered out by further analysis. This shows that pSCNN is an efficient method for identifying compounds in real unknown mixtures.

### 4.6. Translation Invariance for NMR Peaks

In NMR spectra, the chemical shifts of the same compound may vary in different samples because of influences from interactions between components, instruments or the environment. Convolutional neural networks have a translational invariance advantage due to the learned high-level representations from raw signals by their convolutional layers and pooling layers. Therefore, it would be interesting to investigate the relationship between chemical shift variations in NMR spectra and translation invariance of the convolutional neural networks.

**Figure 6:**
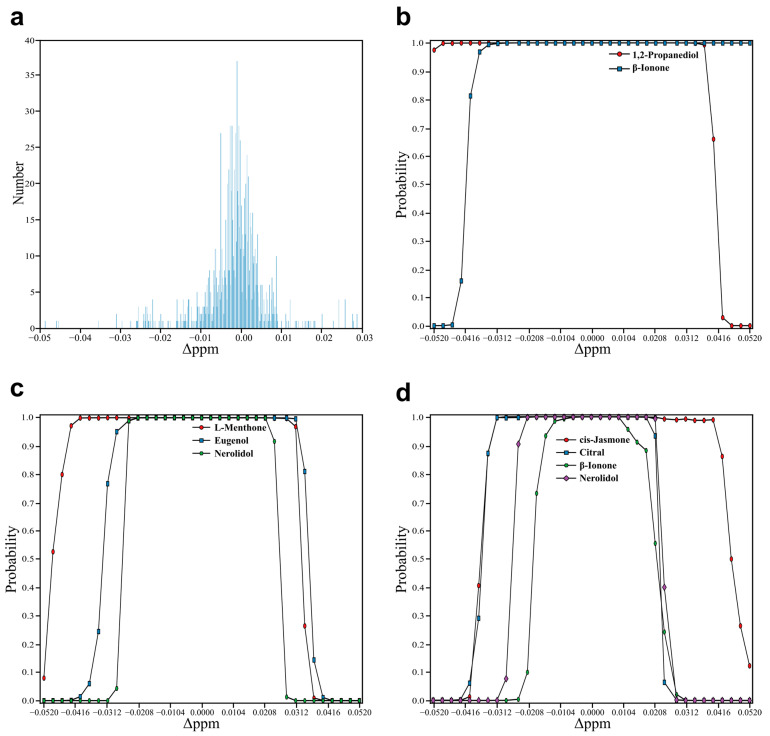
Demonstration of the translation invariance of pSCNN. (**a**) The number of chemical shift variations between all mixtures and their corresponding components. (**b**–**d**) The probabilities of the corresponding components in mixtures predicted by pSCNN for spectral pairs with different chemical shift variations. The results in (**b**–**d**) are the spectral pairs with two, three and four components, respectively.

First, the experimental NMR spectra are used to determine the interval of the chemical shift variations. The chemical shift of active hydrogen is highly correlated with the concentration of the compound because of the hydrogen-bonding interaction, so the chemical shift variations of active hydrogen in different NMR spectra are not taken into account. The chemical shift variations of the deuterated solvent (DMSO-d_6_ 2.50 ppm and HDO 3.33 ppm [79]) are also not considered, as the solvent signal is not the signal of interest. By observing the NMR spectra of all mixtures and their corresponding components, the chemical shift variation of each spectral pair was calculated by the deviation between the chemical shift of the peaks of the mixture and the chemical shift of the peaks for its component. As shown in Figure 6a, the mean value and the standard deviation of the chemical shift variation are −0.0016 and 0.0077, respectively. The obtained interval was (−0.013, 0.010) according to the mean value ±1.5 × standard deviation. This interval contains the chemical shift variations of 90.52% of the characteristic peaks in the spectral pairs.

To generate NMR spectra with different chemical shift variations, the NMR spectra of the mixtures were obtained by the data augmentation method, and their corresponding components were retrieved from the spectral database. The number of components in these mixtures are 2, 3, 4 and 5. Two spectral pairs were generated under each number of component; thus, eight spectral pairs were generated. The information of the augmented spectral pairs is listed in Table S6. A total of 28 spectral pairs were obtained by combining each mixture with its components. For each spectral pair, chemical shifts of the NMR spectrum of its mixture were varied gradually within ±0.052 ppm ranges, and the chemical shifts of the NMR spectra of its components were unchanged. A varied spectral pair was obtained after each chemical shift variation, and 35 varied spectral pairs were obtained for each spectral pair. Thus, 980 spectral pairs with different chemical shift variations were obtained to verify the translation invariance of the pSCNN model. The overall scheme for verifying the translation invariance is shown in Figure S1. For the spectral pair of a specific mixture and a specific component, its varied spectral pairs were fed into the pSCNN model to predict the probabilities of the component in the mixture under different chemical shift variations. The probabilities of the spectral pairs with different chemical shift variations are listed in Table S7. The results of representative spectral pairs are shown in Figure 6b–d, respectively. It can be seen that the minimum interval with probabilities higher than 0.5 (−0.015, 0.015) exceeded the obtained interval (−0.013, 0.010). The results showed that pSCNN can be directly used to compare the two NMR spectra without chemical shift alignment.

## 5. Conclusions

In this study, an end-to-end method for compound identification in mixtures was developed based on a pseudo-Siamese convolutional neural network and ^1^H NMR spectroscopy. Two subnetworks consisting of convolutional layers were chosen to learn the representations from the spectra of pure compounds and the representations from the spectra of mixtures, respectively. The pure compound representation and the mixture representation were concatenated and fed into the dense layers to predict the probability of the compound in the mixture. The data augmentation method was used to generate 22,000 dual inputs from the NMR spectral database of flavor standards, which was randomly divided into the training set, validation set and test set. The performance of pSCNN was evaluated on the test set in terms of the ACC (99.80%), TPR (99.70%) and FPR (0.10%). Furthermore, the proposed method was applied in the flavor mixtures dataset and the additional flavor mixture dataset to benchmark its performance in real mixtures. The performance metrics were ACC = 97.62%, TPR = 96.44% and FPR = 2.29% for the flavor mixtures dataset and ACC = 91.67%, TPR = 100.00% and FPR = 10.53% for the additional flavor mixture dataset. The results show that this method is able to identify components in mixtures accurately. Even in the chemical shift variations up to 0.015 ppm, the trained model can still identify the components in a mixture, which should be attributed to the translation invariance introduced by the convolutional layers and pooling layers in pSCNN. In conclusion, deep learning methods, especially pSCNN, are highly promising approaches to identify compounds in the mixture based on NMR spectroscopy. Due to the flexibility of CNN, the concept of pSCNN can be easily extended to NMR spectral library searching, the verification of complex samples and compound identification with multidimensional NMR spectroscopy. Further, high-field NMR spectrometers and low temperature probes can increase the sensitivity, which may help to analyze more challenging samples and improve the accuracy of the prediction results.

## Figures and Tables

**Table 1:** The accuracy of different pSCNN models on the validation set.

Name of Models	Epoch	The Number of Convolutional Layers *	Learning Rate	ACC
M1	100	6	10^−2^	0.4900
M2	100	6	10^−3^	0.4935
M3	100	6	10^−4^	0.9990
M4	100	6	10^−5^	0.9935
M5	100	5	10^−4^	0.9975
M6	100	7	10^−4^	0.9975
M7	100	8	10^−4^	0.9935
M8	100	9	10^−4^	0.9925
M9	100	10	10^−4^	0.9860

* A max pooling layer whose stride is set to 2 follows a convolutional layer.

**Table 2:** The results of the pSCNN model on the experimental NMR datasets.

Datasets	ACC	TPR	FPR
flavor mixtures dataset	97.62%	96.44%	2.29%
additional flavor mixture dataset	91.67%	100.00%	10.53%

## Data Availability

The source code, model, spectra, manual and tutorial are available at https://www.github.com/yuxuanliao/pSCNN (accessed on 4 May 2022).

## Supplementary Materials

The following supporting information can be downloaded at https://www.mdpi.com/article/10.3390/molecules27123653/s1: Figure S1: Figure of the data augmentation and samples split. Figure S2: Flowchart of the overall scheme for verifying the translation invariance. Table S1: Table of information of 24 flavor standards. Table S2: Table of information of the flavor mixtures. Table S3: Table of information of the additional flavor mixture. Table S4: Table of the detailed results of all mixtures in the flavor mixtures dataset. Table S5: Table of detailed results of the mixture in the additional flavor mixture dataset. Table S6: Table of information of the augmented mixtures. Table S7: Table of probabilities of spectral pairs with different chemical shift variations.

## Sample Availability

Samples of the compounds are available from the authors.
