# Supplementary material for: Deep Learning-Based Method for Compound Identification in NMR Spectra of Mixtures
Source: Molecules. 2022 Jun 7;27(12):3653. doi: 10.3390/molecules27123653 (PMC9227391; doi:10.3390/molecules27123653)
Supplement: Supplementary file 1 [file molecules-27-03653-s001.zip › supporting information.pdf]

molecules

MDPI

Supplementary Materials

Deep Learning-Based Method for Compound Identification in NMR Spectra of Mixtures

Weiwei Wei [1,†], Yuxuan Liao [2,†], Yufei Wang [2], Shaoqi Wang [2], Wen Du [1], Hongmei Lu [2], Bo Kong [1,*], Huawu Yang [3,*] and Zhimin Zhang [2,*]

[1] Technology Center, China Tobacco Hunan Industrial Co., Ltd., Changsha 410014, China; weiww0216@hngytobacco.com (W.W.); duwen0621@hngytobacco.com (W.D.)
[2] College of Chemistry and Chemical Engineering, Central South University, Changsha 410083, China; 212311021@csu.edu.cn (Y.L.); wyf991314@163.com (Y.W.); 8202180822@csu.edu.cn (S.W.); hongmeilu@csu.edu.cn (H.L.)
[3] Flavors and Fragrances Research Institute, Technology Center, China Tobacco Hunan Industrial Co., Ltd., Changsha 410014, China
* Correspondence: kongb0706@hngytobacco.com (B.K.); yanghw0918@hngytobacco.com (H.Y.); zmzhang@csu.edu.cn (Z.Z.)
† These authors contributed equally to this work.

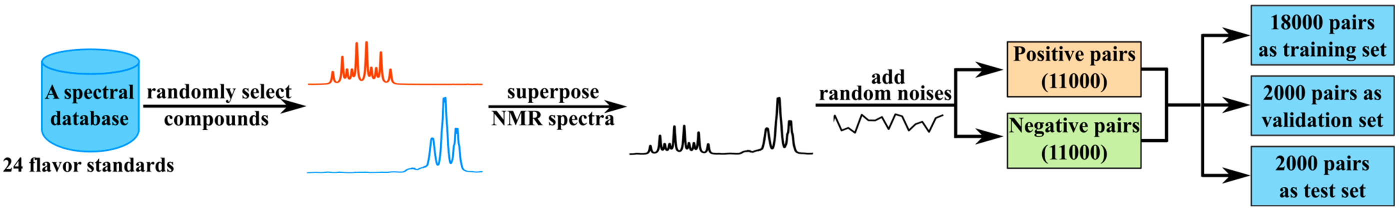

Figure S1. Data augmentation and samples split. A total of 22000 augmented NMR spectral pairs were generated and split to train, validation and test the pSCNN model, and it was obtained by superposing several NMR spectra sampled from a spectral database and the random noises.

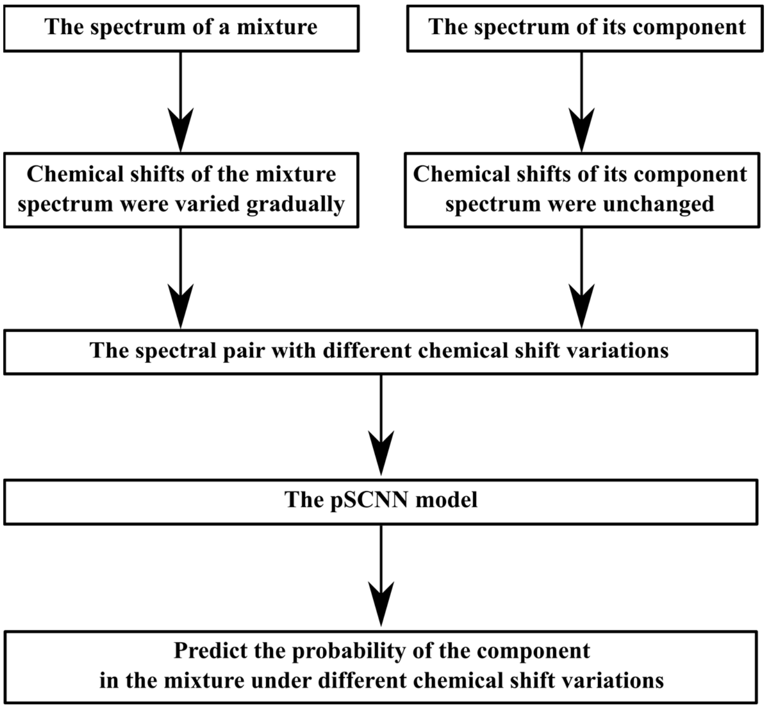

Figure S2. Flowchart of the overall scheme for verifying the translation invariance.

Table S1. The information of 24 flavor standards.

CAS	Name	SMILES
57-55-6	1,2-Propanediol	CC(O)CO
470-82-6	1,8-Cineole	C[C@]12CC[C@H](CC1)C(C)(C)O2
116-53-0	2-Methylbutyric acid	CCC(C)C(O)=O
620-02-0	5-Methylfurfural	[H]C(=O)c1ccc(C)o1
140-11-4	Benzyl acetate	CC(=O)OCc1ccccc1
488-10-8	cis-Jasmone	[H]\C(CC)=C(/[H])CC1=C(C)CCC1=O
5392-40-5	Citral	[H]C(=O)C=C(C)CC\C=C(\C)C
106-23-0	Citronellal	CC(CC\C=C(\C)C)CC=O
97-53-0	Eugenol	COc1cc(CC=C)ccc1O
98-00-0	Furfuryl alcohol	OCc1ccco1
106-24-1	Geraniol	C\C(C)=C\CC\C(C)=C\CO
123-92-2	Isopentyl acetate	CC(C)CCOC(C)=O
503-74-2	Isovaleric acid	CC(C)CC(O)=O
50-21-5	Lactic acid	CC(O)C(O)=O
3681-71-8	Leaf acetate	[H]\C(CC)=C(/[H])CCOC(C)=O
928-96-1	Leaf alcohol	CC\C=C/CCO
115-95-7	Linalyl acetate	C\C(C)=C\CCC(C)(OC(C)=O)C=C
14073-97-3	L-Menthone	CC(C)[C@@H]1CC[C@@H](C)CC1=O
93-15-2	Methyl eugenol	COc1ccc(CC=C)cc1OC
7212-44-4	Nerolidol	C\C(C)=C/CC\C(C)=C\CCC(C)(O)C=C
110-93-0	Sulcatone	C\C(C)=C\CCC(C)=O
14901-07-6	β-Ionone	CC(=O)\C=C\C1=C(C)CCCC1(C)C
706-14-9	γ-Decalactone	CCCCCCC1CCC(=O)O1
104-61-0	γ-Nonanoic lactone	CCCCCC1CCC(=O)O1

Table S2. The information of the flavor mixtures.

Flavor mixture	Formulation	Ratio
F1	Linalyl acetate	0.50
	β-Ionone	0.50
F2	1,2-Propanediol	0.50
	β-Ionone	0.50
F3	Nerolidol	0.50
	Leaf acetate	0.50
F4	β-Ionone	0.33
	Furfuryl alcohol	0.33
	Methyl eugenol	0.33
F5	1,8-Cineole	0.33
	cis-Jasmone	0.33
	5-Methylfurfural	0.33
F6	1,8-Cineole	0.33
	Leaf acetate	0.33
	Citral	0.33
F7	cis-Jasmone	0.33
	Linalyl acetate	0.33
	Eugenol	0.33
F8	Linalyl acetate	0.33
	Geraniol	0.33
	Eugenol	0.33
F9	Linalyl acetate	0.25
	Citral	0.25
	Leaf alcohol	0.25
	2-Methylbutyric acid	0.25
F10	Sulcatone	0.25
	L-Menthone	0.25
	Citronellal	0.25
	Leaf acetate	0.25
F11	β-Ionone	0.25
	Linalyl acetate	0.25
	1,8-Cineole	0.25
	Eugenol	0.25

Table S2. Cont.

Flavor mixture	Formulation	Ratio
F12	Linalyl acetate	0.25
	Leaf acetate	0.25
	Nerolidol	0.25
	L-Menthone	0.25
F13	Linalyl acetate	0.20
	Leaf alcohol	0.20
	γ-Decalactone	0.20
	Isovaleric acid	0.20
	Methyl eugenol	0.20
F14	cis-Jasmone	0.20
	Sulcatone	0.20
	Citral	0.20
	Benzyl acetate	0.20
	Citronellal	0.20
F15	Leaf acetate	0.20
	cis-Jasmone	0.20
	Benzyl acetate	0.20
	Citral	0.20
	Nerolidol	0.20

Table S3. The information of the additional flavor mixture.

Additional flavor mixture	Formulation*	Ratio
U1	β-Ionone	0.20
	2-Methylbutyric acid	0.20
	γ-Nonanoic lactone	0.20
	Citral	0.20
	Leaf alcohol	0.20

* This information was not known at the time of our analysis with the model, and that we were informed of the corresponding components after submitting the result.

Table S4. The detailed results of all mixtures in the flavor mixtures dataset.

No.	Components	Prediction results	ACC(%)	TPR(%)	FPR(%)
F1	Linalyl acetate<br>β-Ionone	Linalyl acetate<br>β-Ionone	100.0	100.0	0.0
F2	1,2-Propanediol<br>β-Ionone	1,2-Propanediol<br>β-Ionone<br>2-Methylbutyric acid	95.83	100.0	4.55
F3	Nerolidol<br>Leaf acetate	Nerolidol<br>Leaf acetate	100.0	100.0	0.0
F4	β-Ionone<br>Furfuryl alcohol<br>Methyl eugenol	β-Ionone<br>Furfuryl alcohol<br>Methyl eugenol	100.0	100.0	0.0
F5	1,8-Cineole<br>cis-Jasmone<br>5-Methylfurfural	1,8-Cineole<br>cis-Jasmone	95.83	66.67	0.0
F6	1,8-Cineole<br>Leaf acetate<br>Citral	1,8-Cineole<br>Leaf acetate<br>Citral	100.0	100.0	0.0
F7	cis-Jasmone<br>Linalyl acetate<br>Eugenol	cis-Jasmone<br>Sulcatone<br>Eugenol<br>Linalyl acetate	95.83	100.0	4.76
F8	Linalyl acetate<br>Geraniol<br>Eugenol	Linalyl acetate<br>Methyl eugenol<br>Eugenol<br>Geraniol	95.83	100.0	4.76
F9	Linalyl acetate<br>Citral<br>Leaf alcohol<br>2-Methylbutyric acid	Linalyl acetate<br>Citral<br>Leaf alcohol<br>Isovaleric acid<br>2-Methylbutyric acid	91.67	100.0	5.0
F10	Sulcatone<br>L-Menthone<br>Citronellal<br>Leaf acetate	Sulcatone<br>L-Menthone<br>Citronellal<br>Leaf acetate	100.0	100.0	0.0

Table S4. Cont.

No.	Components	Prediction results	ACC(%)	TPR(%)	FPR(%)
F11	β-Ionone<br>Linalyl acetate<br>1,8-Cineole<br>Eugenol	β-Ionone<br>Linalyl acetate<br>1,8-Cineole<br>Eugenol<br>Methyl eugenol	95.83	100.0	5.0
F12	Linalyl acetate<br>Leaf acetate<br>Nerolidol<br>L-Menthone	Linalyl acetate<br>Leaf acetate<br>Nerolidol<br>Isovaleric acid<br>L-Menthone	95.83	100.0	5.0
F13	Linalyl acetate<br>Leaf alcohol<br>γ-Decalactone<br>Isovaleric acid<br>Methyl eugenol	Linalyl acetate<br>Leaf alcohol<br>γ-Decalactone<br>γ-Nonanoic lactone<br>Methyl eugenol<br>Isovaleric acid	95.83	100.0	5.26
F14	cis-Jasmone<br>Sulcatone<br>Citral<br>Benzyl acetate<br>Citronellal	cis-Jasmone<br>Sulcatone<br>Citral<br>Benzyl acetate<br>Citronellal	100.0	100.0	0.0
F15	Leaf acetate<br>cis-Jasmone<br>Benzyl acetate<br>Citral<br>Nerolidol	Leaf acetate<br>cis-Jasmone<br>Benzyl acetate<br>Citral<br>Sulcatone	95.83	80.0	0.0

Table S5. The detailed results of the mixture in the additional flavor mixture dataset.

No.	Components	Prediction results	ACC(%)	TPR(%)	FPR(%)
U1	β-Ionone<br>2-Methylbutyric acid<br>γ-Nonanoic lactone<br>Citral<br>Leaf alcohol	β-Ionone<br>γ-Decalactone<br>γ-Nonanoic lactone<br>Citral<br>Leaf alcohol<br>Isovaleric acid<br>2-Methylbutyric acid	91.67	100.00	10.53

Table S6. The information of the augmented mixtures.

Augmented mixture	Formulation	Ratio
A1	cis-Jasmone	0.50
	Leaf alcohol	0.50
A2	1,2-Propanediol	0.50
	β-Ionone	0.50
A3	Citral	0.33
	Citronellal	0.33
	Methyl eugenol	0.33
A4	L-Menthone	0.33
	Eugenol	0.33
	Nerolidol	0.33
A5	cis-Jasmone	0.25
	Citral	0.25
	β-Ionone	0.25
	Nerolidol	0.25
A6	Sulcatone	0.25
	L-Menthone	0.25
	Citronellal	0.25
	Leaf acetate	0.25
A7	γ-Decalactone	0.20
	Leaf alcohol	0.20
	Methyl eugenol	0.20
	β-Ionone	0.20
	Isopentyl acetate	0.20
A8	β-Ionone	0.20
	2-Methylbutyric acid	0.20
	γ-Nonanoic lactone	0.20
	Citral	0.20
	Leaf alcohol	0.20
